# A Unique Case of Atrial Fibrillation Secondary to Squamous Cell Lung Carcinoma

**DOI:** 10.7759/cureus.44965

**Published:** 2023-09-09

**Authors:** Mohammad J Malik, Emily K Wilson, Vijay Bandhakavi

**Affiliations:** 1 Critical Care Medicine, Philadelphia College of Osteopathic Medicine, Valdosta, USA; 2 Critical Care Medicine, South Georgia Medical Center, Valdosta, USA

**Keywords:** thoracic surgery, lung carcinoma, cardiac arrhythmias, lung mass, squamous cell lung carcinoma, atrial fibrillation

## Abstract

Atrial fibrillation (AF) is widely considered to be the most prevalent cardiac arrhythmia with an incidence of roughly 1%-2% in the United States alone. The incidence of AF has been known to increase with advancing age and thus presents a significant burden on healthcare systems across the globe. AF arises as a result of several mechanisms including structural changes that occur in the heart over time. Here, we present a case in which a 63-year-old male with no past medical history except heavy tobacco use presented to the emergency department complaining of shortness of breath. He also endorsed having palpations and a productive cough for several weeks prior to presenting to the emergency department. An EKG revealed AF with a rapid ventricular response. His chest x-ray revealed an irregular opacification of the left lung; however, a chest computed tomography unveiled a left hilar mass extending to the left upper lobe. The mass was causing obstruction of the left upper lobe and encasement of the left main pulmonary artery and left atrium. This case highlights a rare etiology of AF. While many causes of AF have been elucidated, including hypertension and valvular heart disease, a much lesser-known cause includes lung carcinoma resulting in a mass effect on the heart. Representing almost 19% of all cancer deaths, lung cancer is the leading cause of cancer death. Although lung cancer screenings are recommended for certain populations, the majority of lung cancer cases present at an advanced stage, limiting treatment options. Our patient presents a unique case involving a lung mass causing AF due to the mass effect on the left heart. Although the patient had other risk factors for AF including advanced age and cigarette smoking, we propose that due to the anatomical location of his lung mass, his AF was a consequence of the squamous cell carcinoma of the lung. Although the mortality for lung cancer remains high, new treatments, including pembrolizumab, have the potential to drastically alter the way these cancers are treated.

## Introduction

Atrial fibrillation (AF) is widely considered to be the most prevalent cardiac arrhythmia with an incidence of roughly 1%-2% in the United States alone [1]. The incidence of AF has been known to increase with advancing age and thus presents a significant burden on healthcare systems across the globe. AF arises as a result of several mechanisms including structural changes that occur in the heart over time. Hypertension has been well-documented to cause an increased risk of developing AF [2,3]. Long-standing hypertension causes many deleterious effects on the heart including left ventricular hypertrophy resulting in diastolic heart dysfunction [4]. Over time, elevated pressures in the left ventricle can cause left atrial dilation and remodeling which has been known to play a significant role in the development of AF [5]. AF most frequently arises as a result of ectopic beats that originate in the pulmonary veins [6]. The left atrium and pulmonary veins are anatomically continuous structures. As a result, left atrial dilation has major effects on the adjacent pulmonary veins [7]. A much rarer cause of atrial fibrillation includes masses in the mediastinum. Here, we present a case in which a 63-year-old male presented to the emergency department complaining of palpitations, cough, and shortness. Moreover, this article was presented as a poster at the 2023 Philadelphia College of Osteopathic Medicine Research Day on May 9, 2023.

## Case presentation

**Figure 1:**
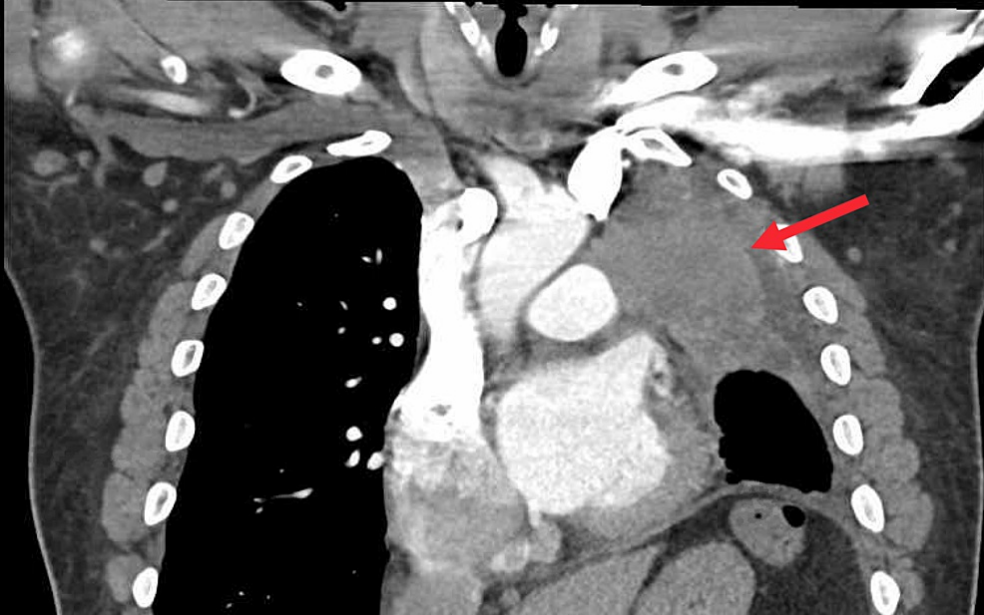
CT scan of the chest demonstrating lung mass compressing on the patient’s left atrium. The arrow indicates the mass.

A 63-year-old male with a past medical history of heavy tobacco use presented to the emergency department complaining of shortness of breath. He also endorsed having palpations and a productive cough for several weeks prior to presenting to the emergency department. An EKG revealed AF with a rapid ventricular response. His chest x-ray revealed an irregular opacification of the left lung; however, a chest computed tomography was obtained which revealed a left hilar mass extending to the left upper lobe. The mass was causing obstruction of the left upper lobe and was encasing the left main pulmonary artery and left bronchus (Figure 1). Subsequent endobronchial ultrasound-guided biopsy revealed squamous cell carcinoma (SCC) of the lung. The patient's atrial fibrillation was treated with metoprolol during his hospitalization. After discharge, the patient followed up with oncology for treatment involving chemotherapy and radiation.

## Discussion

This case highlights a rare etiology of AF. While many causes of AF have been elucidated, including hypertension and valvular heart disease, a much lesser-known cause includes lung carcinoma resulting in a mass effect on the heart. Representing almost 19% of all cancer deaths, lung cancer is the leading cause of cancer death [8,9]. A subtype of lung cancer includes SCC. Epidemiological studies have found that SCC is the most common lung cancer subtype in males and is thought to almost exclusively arise as a result of smoking [10,11]. Although lung cancer screenings are recommended for certain populations, the majority of lung cancer cases present at an advanced stage. and thus, treatment options are limited. Treatment for early-stage SCC of the lung includes surgical resection, whereas treatment for more advanced stages is centered around chemotherapy and, more recently, immune checkpoint inhibitors including pembrolizumab [12,13].

## Conclusions

Our patient presents a unique case involving a lung mass causing AF due to a mass effect on the left heart. Although the patient in this case had other risk factors for AF including advanced age and cigarette smoking, it can be presumed that due to the anatomical location of his lung mass, his AF was a result of his SCC. Although the mortality for lung cancer remains high, new treatments, including pembrolizumab, have the potential to drastically alter the way these cancers are treated.

## References

[1] Kornej J, Börschel CS, Benjamin EJ, Schnabel RB (2020). Epidemiology of atrial fibrillation in the 21st century: novel methods and new insights. Circ Res.

[2] Andrade J, Khairy P, Dobrev D, Nattel S (2014). The clinical profile and pathophysiology of atrial fibrillation: relationships among clinical features, epidemiology, and mechanisms. Circ Res.

[3] Verdecchia P, Reboldi G, Gattobigio R (2003). Atrial fibrillation in hypertension: predictors and outcome. Hypertension.

[4] Dzeshka MS, Shantsila A, Shantsila E, Lip GY (2017). Atrial fibrillation and hypertension. Hypertension.

[5] Andersen JS, Egeblad H, Abildgaard U, Aldershvile J, Godtfredsen J (1991). Atrial fibrillation and left atrial enlargement: cause or effect?. J Intern Med.

[6] Gerstenfeld EP, Callans DJ, Dixit S, Zado E, Marchlinski FE (2003). Incidence and location of focal atrial fibrillation triggers in patients undergoing repeat pulmonary vein isolation: implications for ablation strategies. J Cardiovasc Electrophysiol.

[7] Kim S, Kim YH, Lee SH, Kim JS (2020). Pulmonary vein enlargement as an independent predictor for new-onset atrial fibrillation. J Clin Med.

[8] Dela Cruz CS, Tanoue LT, Matthay RA (2011). Lung cancer: epidemiology, etiology, and prevention. Clin Chest Med.

[9] Cheng TY, Cramb SM, Baade PD, Youlden DR, Nwogu C, Reid ME (2016). The International Epidemiology of Lung Cancer: latest trends, disparities, and tumor characteristics. J Thorac Oncol.

[10] Meza R, Meernik C, Jeon J, Cote ML (2015). Lung cancer incidence trends by gender, race and histology in the United States, 1973-2010. PLoS One.

[11] Barbone F, Bovenzi M, Cavallieri F, Stanta G (1997). Cigarette smoking and histologic type of lung cancer in men. Chest.

[12] Deboever N, Mitchell KG, Feldman HA, Cascone T, Sepesi B (2022). Current surgical indications for non-small-cell lung cancer. Cancers (Basel).

[13] Garon EB, Rizvi NA, Hui R (2015). Pembrolizumab for the treatment of non-small-cell lung cancer. N Engl J Med.

